# Simulation of an Orthodontic System Using the Lingual Technique Based on the Finite Element Method

**DOI:** 10.3390/diagnostics14242832

**Published:** 2024-12-16

**Authors:** Abbas Hazem, Felicia Ileana Mărășescu, Mihaela Jana Țuculină, Dragoș Laurențiu Popa, Ionuț Daniel Geonea, Alexandru Iliescu, Petre Mărășescu, Ioan Ovidiu Gheorghe, Alma Roxana Pitru, Eugen Nicolae Tieranu, Ionela Teodora Dascălu

**Affiliations:** 1 Department of Orthodontics, Faculty of Dental Medicine, University of Medicine and Pharmacy of Craiova, 200349 Craiova, Romania; hazem6070@gmail.com (A.H.); ciuca_felicia@yahoo.com (F.I.M.); marceldascalu@yahoo.com (I.T.D.); 2 Department of Endodontics, Faculty of Dental Medicine, University of Medicine and Pharmacy of Craiova, 200349 Craiova, Romania; popadragoslaurentiu@yahoo.com; 3 Department of Automotive, Transportation and Industrial Engineering, Faculty of Mechanics, University of Craiova, 200478 Craiova, Romania; igeonea@yahoo.com; 4 Department of Oral Rehabilitation, Faculty of Dental Medicine, University of Medicine and Pharmacy of Craiova, 200349 Craiova, Romania; dentalexro@gmail.com; 5 Department of Dental Prosthesis Technology, Faculty of Dental Medicine, University of Medicine and Pharmacy of Craiova, 200349 Craiova, Romania; 6 Doctoral School, Faculty of Medicine, University of Medicine and Pharmacy of Craiova, 200349 Craiova, Romania; ovis45@yahoo.com; 7 Department of Oral Pathology, Faculty of Medicine, University of Medicine and Pharmacy of Craiova, 200349 Craiova, Romania; allma.pitru@umfcv.ro; 8 Department of Internal Medicine-Cardiology, Faculty of Medicine, University of Medicine and Pharmacy of Craiova, 200349 Craiova, Romania; eugen.tiereanu@umfcv.ro

**Keywords:** FEM, archwires, brackets, lingual technique, orthodontic system, internal structures of the teeth

## Abstract

**Backgrounds/Objectives:** The finite element method (FEM) is an advanced numerical technique that can be applied in orthodontics to study tooth movements, stresses, and deformations that occur during orthodontic treatment. It is also useful for simulating and visualizing the biomechanical behavior of teeth, tissues, and orthodontic appliances in various clinical scenarios. The objective of this research was to analyze the mechanical behavior of teeth, tissues, and orthodontic appliances in various clinical scenarios. **Materials and Methods:** For this study, we utilized a model derived from a set of CBCT scans of a 26-year-old female patient who underwent fixed orthodontic treatment using the lingual technique. Through a series of programs based on reverse engineering, we constructed a three-dimensional reconstruction of the teeth and their internal structures. Using the finite element method (FEM), we obtained six simulations of an orthodontic system utilizing the fixed lingual technique, in which we employed brackets made of chrome–nickel or gold, and archwires made of nitinol, gold, or stainless steel. **Results:** The study reveals that although the deformation of the archwires during orthodontic treatment is the same, the forces generated by the three types of archwires on brackets differ. The variation in forces applied to the brackets in the fixed lingual orthodontic technique is essential for customizing orthodontic treatment, as these forces must be precisely controlled to ensure effective tooth movement and prevent overloading of the dental structures. **Conclusions:** The FEM analysis allows for the identification of ideal combinations between the materials used for orthodontic archwires and the materials used for brackets. This ensures that the optimal intensity of forces applied during the fixed lingual orthodontic technique results in desired tooth movements without causing damage to the enamel, dentin, or pulp of the teeth.

## 1. Introduction

The forces produced by the process of mastication, or the forces triggered during orthodontic treatment in patients with dento-maxillary anomalies, lead to the development of tensions that are transmitted through the periodontal ligaments of the upper and lower jaws. In orthodontics, understanding the etiology and distribution of these tensions is crucial for restoring the functional balance of the dento-maxillary apparatus, diagnosing dento-maxillary anomalies, and implementing correct orthodontic treatment [1]. Usually, in orthodontics, two-dimensional imaging techniques (such as panoramic radiographs and teleradiographs) are predominantly used to investigate the structures of the dento-maxillary apparatus. These techniques are still essential in orthodontics due to their accessibility and low radiation dose but offer a limited perspective for the three-dimensional evaluation of the orofacial skeleton. The transition from two-dimensional to three-dimensional imaging techniques using CBCT represents a significant step forward in orthodontic diagnosis and treatment planning [2,3].

In orthodontics, cone-beam computed tomography (CBCT) is useful for providing detailed evaluations of the bony structure and tooth positioning, especially in cases of complex dental or skeletal anomalies. Thus, CBCT has become an indispensable imaging tool in orthodontics, ensuring a more accurate and personalized approach to orthodontic treatments [4,5,6].

Since 1990, various professional associations have imposed certain guidelines for the proper use of CBCT in orthodontics. These associations assert that CBCT should only be utilized in clinical situations where other investigative methods do not provide sufficient information for an accurate diagnosis [4,5,6].

Starting with the standard CBCT of a patient, by using several programs with the help of specific reverse and direct engineering techniques, three-dimensional reconstructions can be made in the virtual environment of the skeleton of the dento-maxillary apparatus and the structure of the patient’s teeth. The components of the fixed orthodontic appliance can also be virtually transposed using these techniques. In this way, FEM can be used to carry out research and simulations that cannot be performed in the patient’s oral cavity.

The finite element method (FEM) is an advanced numerical technique that can also be applied in orthodontics to study tooth displacements and the stresses that occur during orthodontic treatment. The accuracy of the results obtained through this technique demonstrates its use for simulating and visualizing the biomechanical behavior of teeth, tissues, and orthodontic appliances in various clinical scenarios. Additionally, FEM can identify areas at increased risk of bone resorption or other injuries that may occur due to the incorrect application of forces during orthodontic treatment [7,8].

As patients’ esthetic demands during fixed orthodontic treatment become increasingly important, new materials and technologies for manufacturing brackets (both vestibular and lingual) are being developed, offering a balance between functionality and esthetics. Brackets for the lingual technique can be made from gold (a gold–platinum–iridium alloy), ceramic, or stainless steel [8,9,10]. Archwires for fixed orthodontic techniques can be made from gold, stainless steel, or esthetic materials. Orthodontists have a wide range of orthodontic archwires of varying sizes and materials at their disposal. Depending on the requirements of the clinical case, it is essential for orthodontists to select the appropriate archwires to achieve the optimal outcome in orthodontic treatment [11,12,13].

## 2. Objective

The objective of this virtual experimental study was to determine the mechanical behavior of an orthodontic system using the lingual technique for fixed orthodontic treatment. The model includes internal structures such as enamel, dentin, and dental pulp based on a set of CBCT scans from a 26-year-old female patient.

The null hypothesis of the study is that all three types of orthodontic archwires (nitinol, gold, and stainless steel) produce similar forces acting on the bracket components, as the deformations of the archwires are identical in all three scenarios.

## 3. Materials and Methods

This in vitro study was conducted at the Orthodontic Clinic of the Faculty of Dental Medicine, U.M.F. Craiova, and received ethical approval from the Ethics Committee (Ref. No. 152/11.07.2022). This study adhered to U.M.F. Craiova’s ethical guidelines for research involving human subjects, and informed consent was obtained from the patient before the initiation of the study.

Our research was conducted throughout 2023.

The lingual orthodontic technique treatment is used in the treatment of many dento-maxillary anomalies, with the exception of deeply covered occlusion where there is a risk of bracket detachment. For this study, the optimal intensity of the forces that can be applied during therapy with the lingual technique was verified to determine the tooth movements we want without compromising the tooth structure.

Thus, the CBCT of a 26-year-old patient diagnosed with mild dento-alveolar disharmony and mild dental crowding was needed.

Based on the CBCT, a model was created and imported into a software program that utilized mathematical algorithms specific to the finite element method (FEM). The materials and their physical–mechanical properties were defined, as well as the constraints affecting the entire analyzed system and the force-loading system, which was determined in a prior study [14,15].

After running the simulation, maps showing displacements, deformations, and stresses were obtained [16,17,18,19]. The results were organized into comparative diagrams, which led to discussions and conclusions.

The finite element method was used in conjunction with a Hewlett Packard graphics workstation to define the virtual models and evaluate the mechanical behavior of the analyzed system. Data analysis, 3D model generation, and certain FEM-based simulations were performed on a series of desktop computers and a Lenovo laptop.

For converting CBCT images into geometric structures, specifically point cloud data, InVesalius 3.1 (CTI, Campinas, Brazil) was initially used [20,21]. The geometry obtained from InVesalius was processed and transformed into closed watertight surfaces using Geomagic and Geomagic 2014 for SolidWorks software (3D Systems, Rock Hill, SC, USA) [22,23]. This software uses reverse engineering techniques and methods to transform anatomical structures into three-dimensional geometries.

The conversion of watertight surfaces into virtual solids and the modeling of orthodontic archwires and brackets was performed using SolidWorks 2022 (Dassault Systèmes, Velizy-Villacoublay, France), a CAD (Computer-Aided Design) program dedicated to direct engineering [24,25]. This software enables the direct modeling of metallic components in parameterized versions composed of virtual solids.

Then, Ansys Workbench 2019 (Ansys, Inc., Canonsburg, PA, USA) was used to determine the mechanical behavior of the analyzed structures. Ansys software simulates the mechanical action of orthodontic wire- and bracket-type elements on anatomical structures using techniques specific to the finite element method.

To generate teeth models, jawbones, and internal dental anatomy, reverse engineering methods were employed. Direct engineering methods were used to model the orthodontic archwires and brackets, and with the help of the material resistance and elasticity theory, the forces acting on the bracket components were determined. To analyze the mechanical behavior of the system under investigation, the finite element method was applied [26].

**Figure 1:**
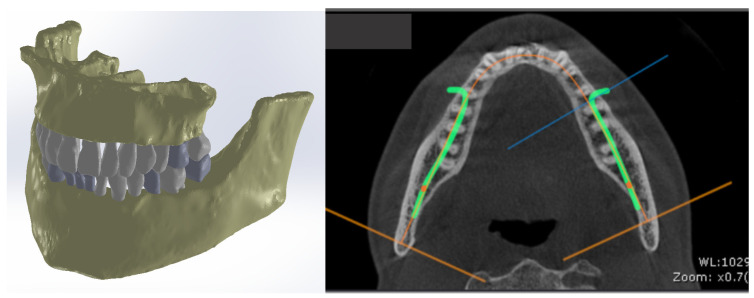
CBCT scan of the patient and the initial model of the orthodontic system.

Subsequently, after modeling the internal structure of several teeth, these models were integrated into the orthodontic system, where lingual fixed orthodontic treatment was used (Figure 1).

**Figure 2:**
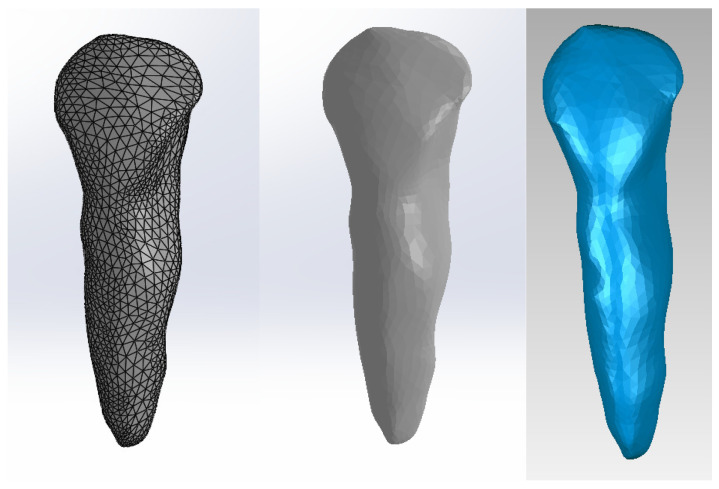
Initial model of canine 1.3: two views in SolidWorks and one view in Geomagic.

To obtain the internal dental structure, we used techniques specific to reverse engineering. For canine 1.3, we used the model shown in Figure 2.

**Figure 3:**
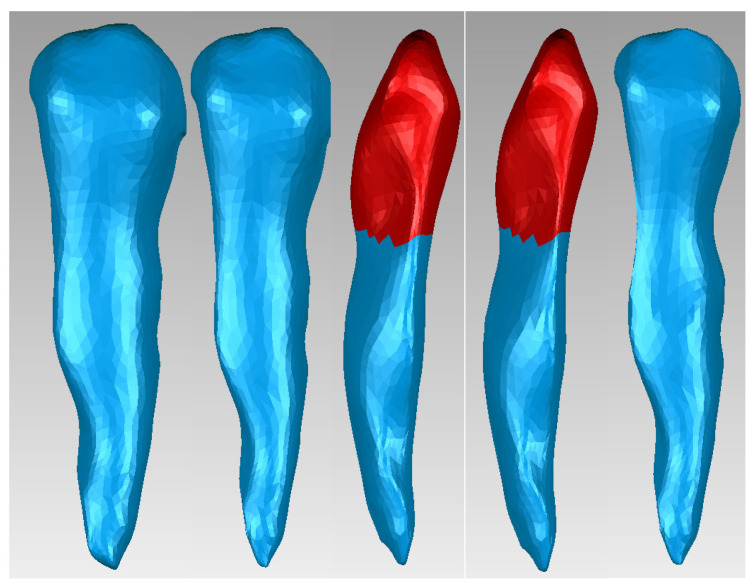
Offset stages applied to the dentin model of canine 1.3.

To obtain the dentin model of canine 1.3 in Geomagic, we used offset techniques applied to the entire model or selected surfaces. Figure 3 presents the stages of using these techniques. The surfaces to which these techniques were applied are red colored.

**Figure 4:**
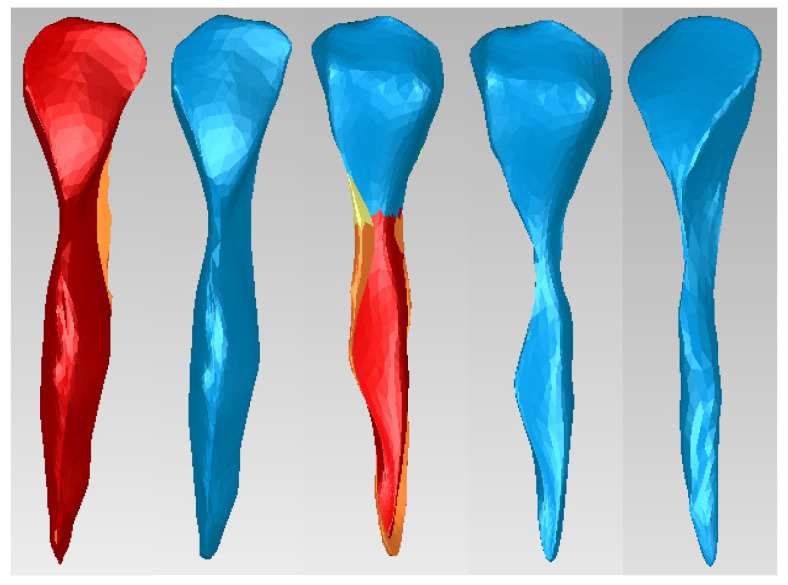
Offset stages applied to the dental pulp model of canine 1.3.

Similar techniques were applied to the dentin model to obtain the dental pulp model of canine 1.3, as shown in Figure 4. The surfaces to which these techniques were applied are red colored.

**Figure 5:**
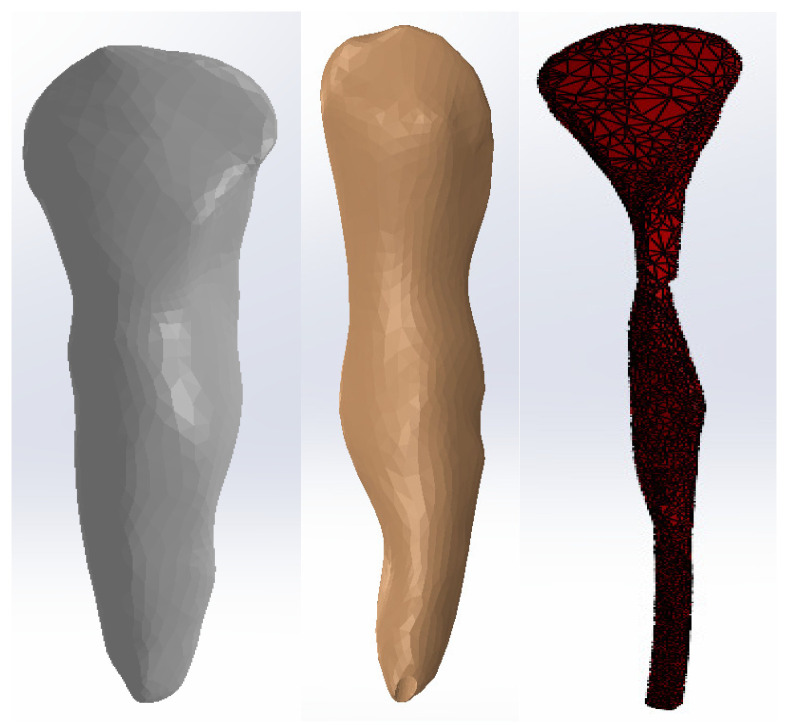
Models of the enamel, dentin, and pulp of canine 1.3 in SolidWorks.

Figure 5 presents the models of enamel, dentin, and dental pulp for canine 1.3 in SolidWorks.

**Figure 6:**
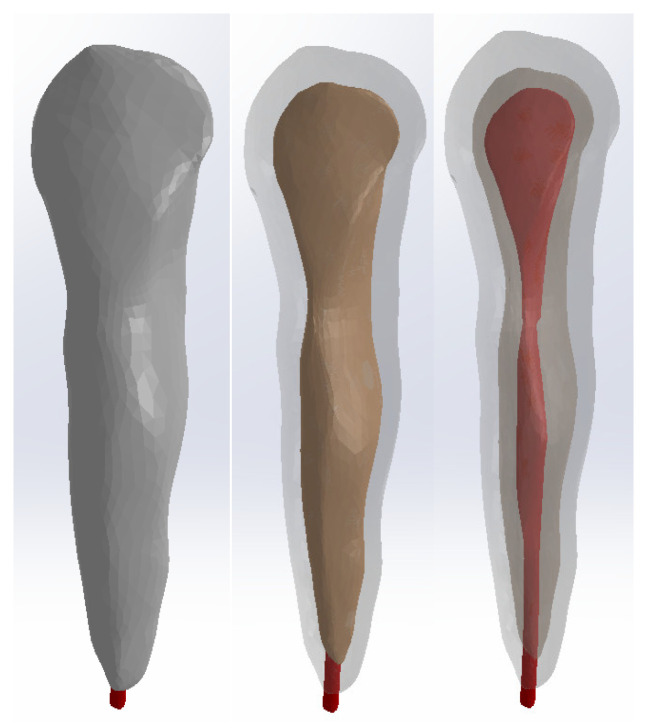
Model of canine 1.3: three views with different degrees of transparency.

We superimposed these models using common coordinate systems for each model. The dentin and pulp models were subtracted volumetrically from the enamel model, and the pulp model was subtracted volumetrically from the dentin model. Figure 6 presents the model of canine 1.3.

Using similar techniques, we obtained models of teeth 1.1, 2.3, 4.3, 2.2, 3.1, 4.2, 1.7, 2.6, 3.7, 4.8, 1.5, 2.4, and 3.5.

**Figure 7:**
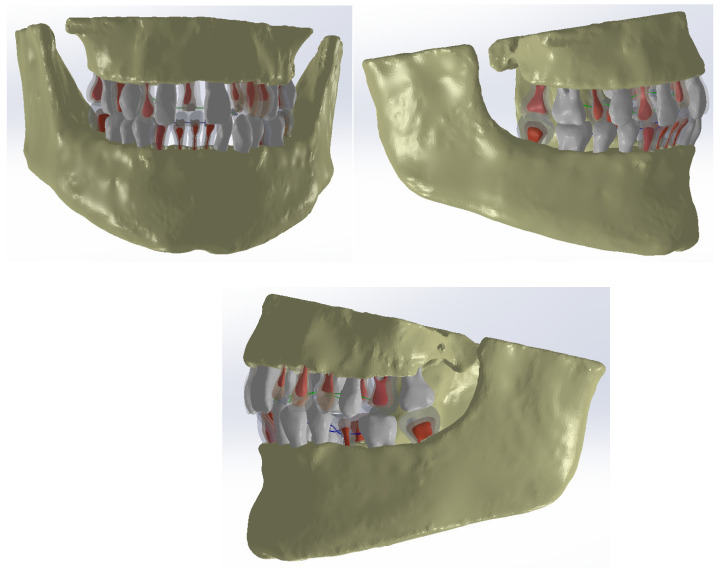
Model of the studied orthodontic system.

These models were incorporated into the orthodontic system model. Figure 7 shows the orthodontic system model with certain structures made transparent.

We obtained six simulations based on the finite element method:

- An orthodontic system based on a nitinol orthodontic archwire and brackets made of a chromium–nickel alloy;
- An orthodontic system based on a nitinol orthodontic archwire and brackets made of gold;
- An orthodontic system based on a gold orthodontic archwire and brackets made of a chromium–nickel alloy;
- An orthodontic system based on a gold orthodontic archwire and brackets made of gold;
- An orthodontic system based on a stainless steel orthodontic archwire and brackets made of a chromium–nickel alloy;
- An orthodontic system based on a stainless steel orthodontic archwire and brackets made of gold.

The model of the analyzed orthodontic system was divided into a structure containing 1.428.581 finite elements and 390.014 nodes. In this model, the orthodontic archwires were removed and replaced with the forces they exert on the bracket elements.

In the Engineering Data module of Ansys Workbench, the material and their physical–mechanical properties were entered or selected from the specialized literature, as shown in Table 1 [14,15,17,18].

Thus, we found three situations in which the forces acting on the bracket elements can be calculated:

1. The orthodontic archwire is made of nitinol (E = 3.45 × 10^10^ Pa);
2. The orthodontic archwire is made of gold (E = 7.8 × 10^10^ Pa);
3. The orthodontic archwire is made of stainless steel (E = 2 × 10^11^ Pa).

For the simulations in which the orthodontic archwire is made of nitinol, the resulting maps and the values of the forces applied in this study can be found in Table 2 (for the maxilla and mandible) and correspond to values found in the specialized literature [27].

For simulations where the orthodontic archwire is made of gold, Table 3 presents the force values in Table 3 (for the maxilla and mandible).

Table 4 presents the force values on the maxilla and mandible for simulations where the orthodontic archwire is made of stainless steel.

## 4. Results

The values of forces applied to the analyzed orthodontic system based on the lingual orthodontic treatment technique were analyzed using six types of simulations [27,28,29,30,31,32,33,34,35,36,37].

**Figure 8:**
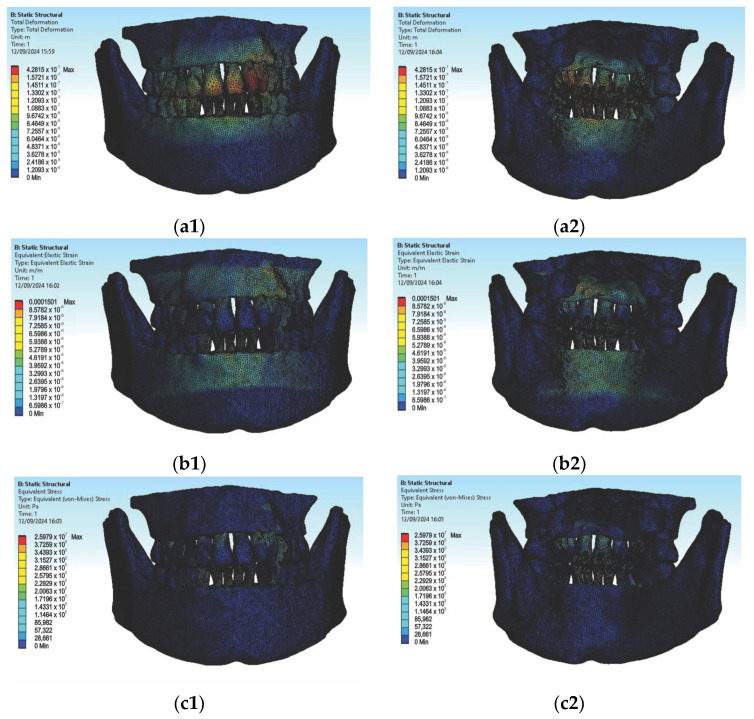
Displacement maps (**a1**,**a2**), deformation maps (**b1**,**b2**), and stress maps (**c1**,**c2**) for nitinol orthodontic archwires and chromium–nickel alloy brackets [27].

In the first simulation, we used orthodontic archwires made of nitinol and bracket components made of a chromium–nickel alloy, and we obtained the following maps (Figure t.25) of displacements (Figure 8 (a1,a2)), deformations (Figure 8 (b1,b2)), and stresses (Figure 8 (c1,c2)).

**Figure 9:**
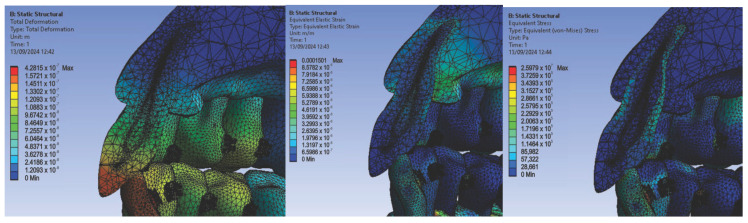
Result maps for a section through incisor 1.1.

Figure 8 presents the displacement maps for the analyzed orthodontic system using a nitinol orthodontic archwire and bracket components made of a chromium–nickel alloy. In contrast, Figure 9 shows the result maps for a section through incisor 1.1. The same procedure was applied to the other teeth (2.3, 4.3, 2.2, 3.1, 4.2, 1.7, 2.6, 3.7, 1.8, 1.5, 2.4, and 3.5) [27].

**Figure 10:**
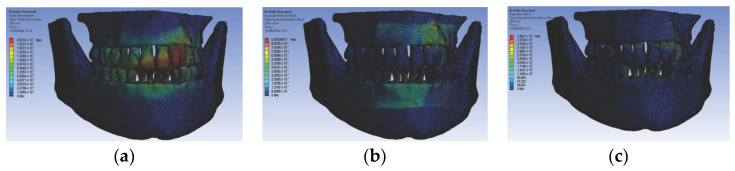
Maps of displacements (**a**), deformations (**b**), and stresses (**c**) for nitinol orthodontic archwires and gold brackets.

**Figure 11:**
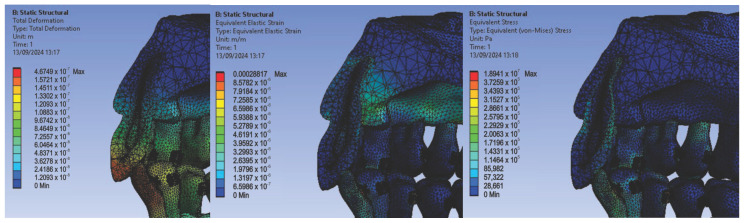
Result maps for a section through incisor 1.1.

In the second simulation, we used orthodontic archwires made of nitinol and bracket components made of gold, obtaining the following maps of displacements (Figure 10a), deformations (Figure 10b), and stresses (Figure 10c) and result maps for a section through incisor 1.1 (Figure 11). We performed a similar process for the other teeth (2.3, 4.3, 2.2, 3.1, 4.2, 1.7, 2.6, 3.7, 1.8, 1.5, 2.4, and 3.5).

**Figure 12:**
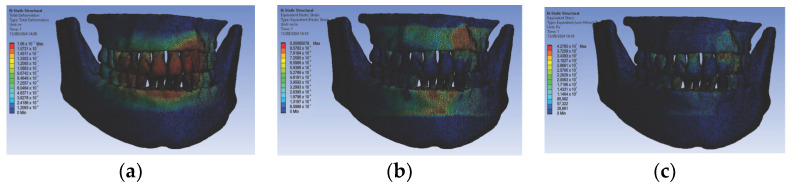
Maps of displacements (**a**), deformations (**b**), and stresses (**c**) for gold orthodontic archwires and gold brackets.

**Figure 13:**
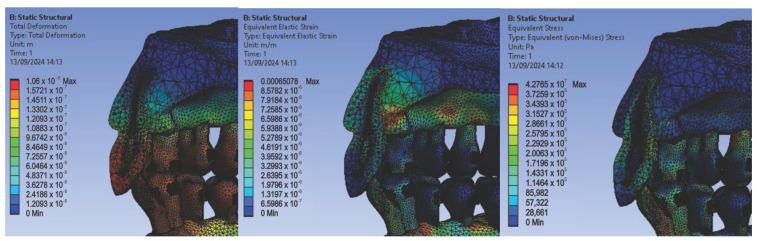
Result maps for a section through incisor 1.1.

In the third simulation, we used gold orthodontic archwires and gold bracket components. As a result of the analysis, we created the following displacement maps (Figure 12a), deformation maps (Figure 12b), and stress maps (Figure 12c), along with result maps for a section through incisor 1.1 (Figure 13). We conducted simulations in the same manner for the teeth 2.3, 4.3, 2.2, 3.1, 4.2, 1.7, 2.6, 3.7, 1.8, 1.5, 2.4, and 3.5.

**Figure 14:**
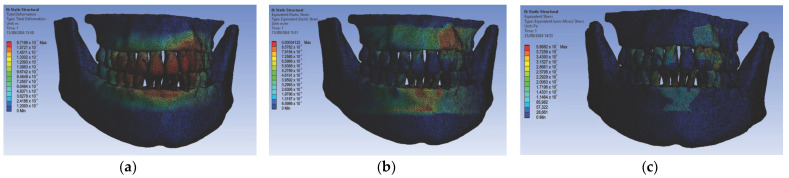
Maps of displacements (**a**), deformations (**b**), and stresses (**c**) for orthodontic archwires made of gold and nickel–chromium alloy brackets.

**Figure 15:**
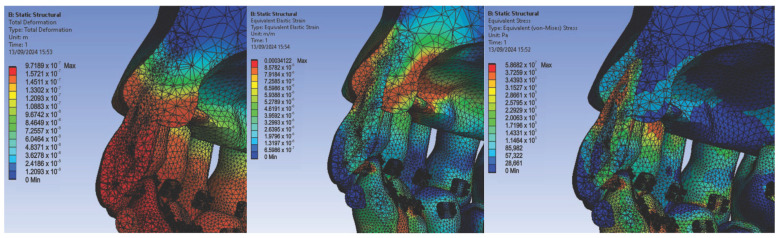
Result maps for a section through incisor 1.1.

We continued with the simulation using orthodontic archwires made of gold and nickel–chromium alloy bracket components, obtaining the following displacement, deformation, and stress maps (Figure 14) and result maps for a section through incisor 1.1 (Figure 15). We followed the same procedure for the other teeth: 2.3, 4.3, 2.2, 3.1, 4.2, 1.7, 2.6, 3.7, 1.8, 1.5, 2.4, and 3.5.

**Figure 16:**
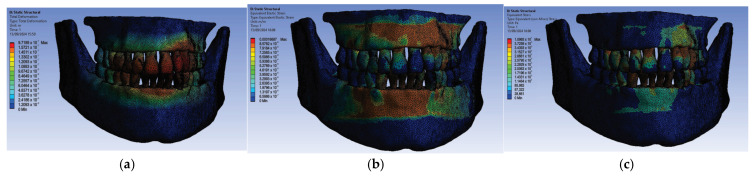
Maps of displacements (**a**), deformations (**b**), and stresses (**c**) for stainless steel orthodontic archwires and gold brackets.

**Figure 17:**
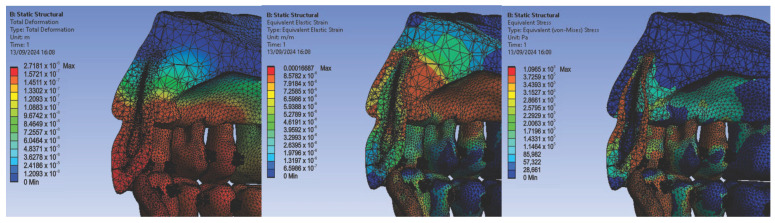
Result maps for a section through incisor 1.1.

Next, we conducted simulations using stainless steel orthodontic archwires and gold bracket components, determining the following displacement, deformation, and stress maps (Figure 16) and result maps for a section through incisor 1.1 (Figure 17). We followed a similar approach with teeth 2.3, 4.3, 2.2, 3.1, 4.2, 1.7, 2.6, 3.7, 1.8, 1.5, 2.5, and 3.5.

**Figure 18:**
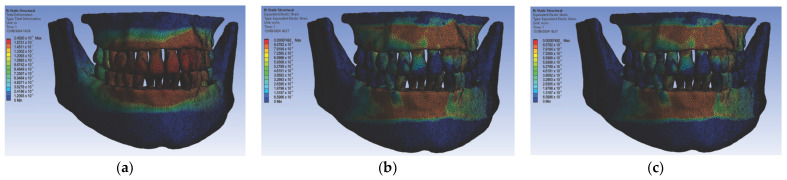
Maps of displacements (**a**), deformations (**b**), and stresses (**c**) for stainless steel orthodontic archwires and nickel–chromium alloy brackets.

**Figure 19:**
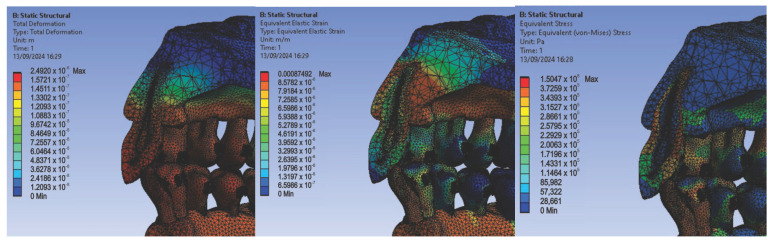
Result maps for a section through incisor 1.1.

Then, we conducted simulations using stainless steel orthodontic archwires and nickel–chromium alloy brackets, obtaining displacement, deformation, and stress maps, as well as result maps for a section through 1.1 (Figure 18 and Figure 19). We followed the same protocol with teeth 2.3, 4.3, 2.2, 3.1, 4.2, 1.7, 2.6, 3.7, 1.8, 1.5, 2.5, and 3.5.

After completing the six simulations, we performed comparative analyses of the results of the simulations on the orthodontic system based on the lingual technique, using different materials for the orthodontic archwire and bracket elements.

**Figure 20:**
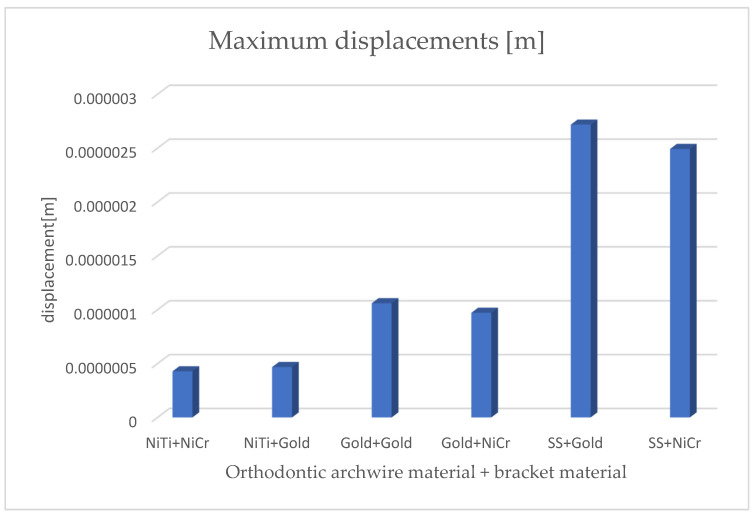
Comparative diagram of maximum displacements.

Figure 20 presents the comparative diagram of the maximum displacements for the six simulations using the finite element method.

By analyzing the comparative diagrams obtained based on the maximum values from the result maps, we found the following:

- The minimum displacements were observed when the orthodontic archwire was made of nitinol and the bracket components were made of a nickel–chromium alloy;
- The maximum displacements were observed when the orthodontic archwire was made of stainless steel and the bracket components were made of gold;
- The minimum deformations were observed when the orthodontic archwire was made of nitinol and the bracket components were made of a nickel–chromium alloy;
- The maximum deformations were observed when the orthodontic archwire was made of stainless steel and the bracket components were made of gold;
- The minimum stresses were observed when the orthodontic archwire was made of nitinol and the bracket components were made of gold;
- The maximum stresses were observed when the orthodontic archwire was made of stainless steel, and the bracket components were made of a nickel–chromium alloy.

**Figure 21:**
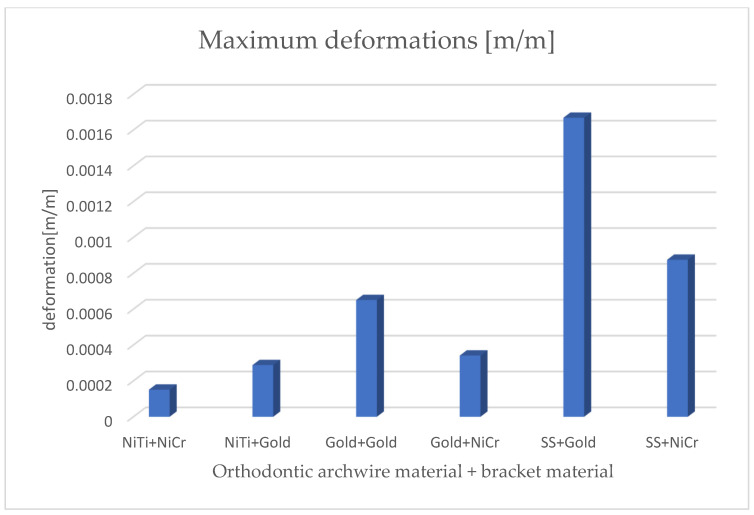
Comparative diagram of maximum deformations.

Figure 21 presents the comparative diagram of maximum deformations for the six simulations using the finite element method.

**Figure 22:**
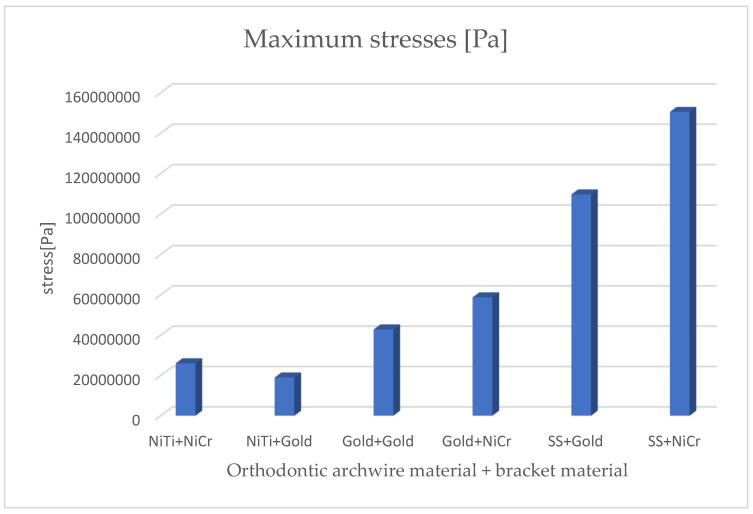
Comparative diagram of maximum stresses.

Figure 22 presents the comparative diagram of maximum stresses for the six simulations using the finite element method.

## 5. Discussion

In accordance with the null hypothesis (h0), all three orthodontic archwires produce the same forces on the bracket components. However, this null hypothesis is rejected by the results of this study, as the forces are different, even though the deformations of the archwires are the same.

In this study, we utilized both inverse and direct engineering techniques to generate models of the internal structures of the teeth. Starting from the external dental geometry, we used offset techniques in the InVesalius program to define the models of the dentin and dental pulp. These models were then loaded and superimposed in the SolidWorks program. Subsequently, a volumetric subtraction method was employed to obtain the cavities within the dental enamel and dentin. These quasi-anatomical structures were overlaid on the models obtained in the previous study, while the initial models were suppressed. As a result, verification sections were made for some of the teeth (at least one from each category).

We then conducted six simulations grouped into three sets, using orthodontic archwires made of nitinol, gold, and stainless steel, paired with two types of bracket components made of a nickel–chromium alloy and gold [27,28,29,30,31,32,33,34,35].

Utilizing mathematical formulas for the forces acting on each bracket, coupled with virtual measurements, we obtained the force system acting on the orthodontic model using the lingual technique for each of the six studied situations. These systems depend on the material used for the orthodontic archwire, as determined by its Young’s modulus (E) [27].

The minimum deformations of the archwire in the lingual technique that do not affect the tooth structure were achieved when using nitinol archwires and Ni-Cr alloy brackets. The minimum stresses to which the orthodontic archwire is subjected in the lingual technique, without causing changes in the tooth structure, result from using nitinol archwires and gold brackets.

The results that we obtained are similar to those of other studies previously conducted [27,34,35].

Orthodontic stainless steel archwires possess multiple advantageous properties. Due to their high modulus of elasticity and elevated yield strength, the archwire can withstand large elastic deformations and repeatedly return to its original shape. The archwire also has excellent corrosion resistance, making it ideal for use in a salivary environment. Stainless steel is well tolerated by the body, offering good biocompatibility. Additionally, it has excellent formability, making it easy to manufacture archwires in various shapes and sizes. Therefore, these archwires provide both strong resistance and great flexibility [36,37,38,39,40,41].

In our study, the maximum displacements, deformations, and stresses were observed with stainless steel archwires, confirming the properties and advantages of using these archwires. However, with the advent of nitinol archwires, stainless steel archwires have seen a reduction in use. According to some studies, one reason for this is that stainless steel archwires have only about one-third to half the resistance and deflection range compared to NiTi archwires. Nevertheless, clinical studies suggest that there are no significant differences in performance between these archwires during fixed orthodontic treatment [36,38,42,43,44].

Nitinol archwires are made from Ni-Ti alloys and possess remarkable properties due to their shape memory and superelasticity, which make them essential for orthodontic archwire fabrication. The shape memory effect occurs due to the “thermoelastic martensitic transformation” of the material (the material can be deformed in one crystalline phase) and transforms back to the austenitic phase (returns to its original shape when heated). This phenomenon occurs within a specific temperature range. Thus, regardless of the degree of deformation or bending of the wire, superelasticity ensures that it maintains a constant force on the teeth during treatment. This allows for efficient and controlled tooth movement without causing discomfort to the patient [36,38,42,43,44].

In our study, the nitinol archwires demonstrated minimal displacements and deformations compared to stainless steel archwires.

Gold archwires are also used in lingual orthodontic treatment, although their use is less common due to their high cost [10]. Gold brackets, often employed in lingual orthodontics, offer not only esthetic advantages but also benefits related to biocompatibility and safety. The Incognito™ technology, introduced in 2004, involves the use of lingual brackets made from a high-gold-content alloy without nickel. This material reduces the risk of oral mucosal inflammation and allergic reactions. Despite these advantages, gold brackets are used less frequently due to their higher cost [10,45,46,47].

Cr-Ni brackets are currently the most widely used due to their affordability, durability, and remarkable mechanical properties. These brackets offer increased rigidity, corrosion resistance, biocompatibility, and durability and are cost-effective [10,48,49,50].

During fixed orthodontic treatment, maintaining oral hygiene is essential to prevent carious lesions and periodontal diseases. Sodium fluoride or sodium lauryl sulfate in toothpaste may promote the corrosion of stainless steel brackets by releasing Ni and Cr ions. Toothpaste containing sodium fluoride can exacerbate the corrosion of stainless steel brackets and archwires, releasing nickel and chromium ions. The release of Ni and Cr ions can lead to cytotoxicity, hypersensitivity, and potentially carcinogenic effects.

It is important to carefully choose toothpaste during fixed orthodontic treatment. The corrosion of brackets and archwires increases friction between them and reduces the mechanical strength of the brackets. This prolongs the duration of orthodontic treatment and negatively affects its quality and efficiency [51,52,53,54,55,56].

Recent advancements in improving the quality of alloys used to manufacture orthodontic archwires have broadened the options available to orthodontists for selecting appropriate materials to enhance the effectiveness of treatment. However, no material is universally ideal for all phases of orthodontic treatment. The ongoing development of new alloys for orthodontic brackets and archwires is expected to improve their performance, potentially reducing treatment duration and patient discomfort [28,29,57,58,59].

Studies in the literature have shown that during orthodontic treatment, the materials used for fixed appliance archwires and brackets can degrade due to corrosion in the oral cavity environment. Corrosion is an electrochemical process where metals used in archwires and brackets release metal ions. In the presence of dental plaque or fluctuations in salivary pH, these ions can lead to discoloration, enamel demineralization, hypersensitivity, pain, and gingival inflammatory reactions [57,58,59,60,61,62,63].

The novelty of the study lies in the use of non-invasive methods applied to virtual models that are nearly identical to those of a real patient. By employing the finite element method (FEM), it is possible to analyze the biomechanical behavior of orthodontic appliances in various clinical scenarios. Through the comparison of these results, an optimal medical strategy can be determined for addressing specific pathologies.

Study Limitations: The study is limited to analyzing the biomechanical behavior of a single patient. We focused only on orthodontic systems based on nitinol, gold, and stainless steel archwires and a Cr-Ni alloy and gold brackets. Other materials were not included in this analysis.

Recommendations for Future Research: Future studies should include more virtual models from multiple patients. The results of these studies should be compared with those already obtained from the analyzed models. Additionally, future research should include other materials used in lingual orthodontics or new materials that may emerge.

## 6. Conclusions

The variation in forces applied to brackets in lingual fixed orthodontic treatment is essential for personalizing the treatment, as these forces must be precisely controlled to ensure effective tooth movement and to prevent overloading of dental structures.

The results obtained from this study can be used to identify ideal combinations of orthodontic archwire materials and bracket materials, ensuring that the optimal force intensity applied during lingual orthodontic treatment leads to the desired tooth movements without causing damage to the enamel, dentin, or dental pulp.

Additionally, orthodontic strategies can be developed based on different treatment durations, using various materials that are customized for each patient. This approach allows for tailored treatment plans that enhance the efficiency and safety of lingual orthodontics. The nitinol archwires demonstrated minimal displacements and deformations compared to stainless steel archwires.

By eliminating many of the clinical difficulties present in conventional techniques and providing a high degree of esthetic and functional satisfaction, the lingual technique is an excellent solution for patients who want discreet and effective orthodontic treatment.

## Figures and Tables

**Table 1:** Materials and their physical–mechanical properties used in the simulations [11,12,14,15].

Component	Material	Density [Kg/m^3^]	Young’s Modulus [Pa]	Poisson’s Ratio
**Teeth**	Enamel	2.958	7.79 × 10^10^	0.3
**Teeth**	Dentin	2.140	1.76 × 10^10^	0.25
**Teeth**	Dental pulp	1.000	1.75 × 10^9^	0.4
**Maxilla, mandible**	Bone	1.400	1 × 10^10^	0.31
**Orthodontic archwire**	Nitinol	6.450	8.3 × 10^7^	0.33
**Bracket elements**	Ni + Cr alloy	8.500	2.1 × 10^11^	0.31
**Orthodontic archwire**	Stainless steel	7.750	1.93 × 10^11^	0.31
**Orthodontic archwire, bracket elements**	Gold	19.000	7.8 × 10^10^	0.42

**Table 2:** Forces on the bracket elements located on the maxilla and mandible (orthodontic archwire made of nitinol) [24].

Forces on the Bracket Elements [N]											
**F16**	**F15**	**F14**	**F13**	**F12**	**F11**	**F21**	**F22**	**F23**	**F24**	**F25**	**F26**
0.181	0.656	0.529	0.298	0.339	0.123	0.307	1.205	1.568	1.049	0.411	0.124
**F46**	**F45**	**F44**	**F43**	**F42**	**F41**	**F31**	**F32**	**F33**	**F34**	**F35**	**F36**
0.096	0.459	0.694	0.293	1.101	1.443	1.493	1.051	0.454	0.365	0.436	0.12

**Table 3:** Forces on the bracket elements located on the maxilla and mandible (orthodontic archwire made of gold).

Forces on the Bracket Elements [N]											
**F16**	**F15**	**F14**	**F13**	**F12**	**F11**	**F21**	**F22**	**F23**	**F24**	**F25**	**F26**
0.410	1.485	1.196	0.674	0.767	0.280	0.695	2.724	3.546	2.372	0.929	0.284
**F46**	**F45**	**F44**	**F43**	**F42**	**F41**	**F31**	**F32**	**F33**	**F34**	**F35**	**F36**
0.219	1.037	1.569	0.664	2.489	3.264	3.376	2.377	1.027	0.826	0.987	0.272

**Table 4:** Forces on the bracket elements located on the maxilla and mandible (orthodontic archwire made of stainless steel).

Forces on the Bracket Elements [N]											
**F16**	**F15**	**F14**	**F13**	**F12**	**F11**	**F21**	**F22**	**F23**	**F24**	**F25**	**F26**
1.053	3.808	3.067	1.729	1.968	0.718	1.782	6.985	9.092	6.083	2.384	0.729
**F46**	**F45**	**F44**	**F43**	**F42**	**F41**	**F31**	**F32**	**F33**	**F34**	**F35**	**F36**
0.562	2.661	4.023	1.703	6.382	8.369	8.657	6.097	2.635759	2.12	2.531	0.699

## Data Availability

The authors declare that the data from this research are available from the corresponding authors upon reasonable request.
